# The Distribution and Sustainable Utilization of Buckwheat Resources under Climate Change in China

**DOI:** 10.3390/plants10102081

**Published:** 2021-09-30

**Authors:** Wen Wen, Zhiqiang Li, Jirong Shao, Yu Tang, Zhijun Zhao, Jingang Yang, Mengqi Ding, Xuemei Zhu, Meiliang Zhou

**Affiliations:** 1 College of Environmental Sciences, Sichuan Agricultural University, Chengdu 611130, China; w.wen@cml.leidenuniv.nl (W.W.); LiZQ0126@outlook.com (Z.L.); 2 Institute of Crop Sciences, Chinese Academy of Agricultural Sciences, Beijing 100081, China; dingmengqi@caas.cn; 3 Institute of Environmental Sciences (CML), Leiden University, Box 9518, 2300 RA Leiden, The Netherlands; 4 Xichen Intelligent Agricultural Technology Co., Ltd., Chengdu 611130, China; shaojr007@163.com; 5 College of Life Sciences, Sichuan Agricultural University, Ya’an 625014, China; 6 Department of Tourism, Sichuan Tourism University, Chengdu 610100, China; ty23651@sina.com; 7 Institute of Archaeology, Chinese Academy of Social Sciences, Beijing 100010, China; zjzhao@cass.org.cn (Z.Z.); jingangyang@yeah.net (J.Y.); 8 Department of Crop Science, College of Agriculture & Life Sciences, Chungnam National University, Yuseong-gu, Daejeon 305-754, Korea

**Keywords:** buckwheat, climate change, biodiversity, MaxEnt, potentially suitable area

## Abstract

Buckwheat is a promising pseudo cereal and its cultivation history can be traced back to thousands of years ago in China. Nowadays, buckwheat is not only an ordinary crop but also a symbol of healthy life because of its rich nutritional and pharmacological properties. In this research, the current suitable areas of 19 wild buckwheat species were analyzed by the MaxEnt model, which proved that southwestern China was the diversity center of buckwheat. Their morphological characteristics and geographical distribution were analyzed for the first time. In addition, it was found that the change of buckwheat cultivation in three periods might be related to the green revolution of main crops and national policies. Meanwhile, the Sustainable Yield Index (SYI) value of buckwheat in China was the lowest from 1959 to 2016. Through the MaxEnt model, the potentially suitable areas of wild buckwheat would contract while cultivated buckwheat would expand under climate change. Accordingly, the diversity of wild buckwheat will decrease. Therefore, it is necessary to protect buckwheat resources as much as possible to strengthen the development and utilization of buckwheat resources. Moreover, the promotion of buckwheat diversity will be an important trade-off between food security, population growth, and land use under climate change.

## 1. Introduction

Buckwheat, belongs to the genus *Fagopyrum* (*Polygonaceae*), used as an important groat cereal and medicinal plant for many years. There are 21 species of genus *Fagopyrum* in total, consisting of nineteen wild species and two cultivated species including *Fagopyrum esculentum* Moench. and *Fagopyrum tataricum* (L.) Gaertn (Table 1) [1]. Although there were still many debates about the center of buckwheat′s origin, many pieces of evidence showed that its origin was in southwestern China, in which buckwheat wild relatives’ resources are abundant [2,3,4]. Moreover, buckwheat has been cultivated in China for thousands of years. In ancient times, buckwheat was grown due to its short growth period, high adaptability, and resistance to barren conditions, which increased its multiple cropping index and had great benefit against disaster prevention [5]. In addition, buckwheat consumption and cultivation have been increasingly emphasized due to its healing effects and nutritional value. Buckwheat plants and groats are rich in flavonoids such as rutin, orientin, vitexin, quercetin, isovitexin, and isoorientin [6,7,8]. Its balanced amino acid content and richness in both lysine and arginine results in a high nutritional value for buckwheat in comparison with other crops [7,8]. Therefore, there is a need for buckwheat resources to be collected for further development and utilization.

Species distribution models (SDMs) are mainly based on occurrence records and environmental variables, which estimate the species niche as well as reflect the suitable habitat distribution of species in the form of probability [9,10]. SDMs mainly include the BIOCLIM [11], Ecological Niche Factor Analysis (ENFA) [12], Generalized Additive Model (GAM) [13], Generalized Linear Model (GLM) [14], Maximum Entropy (MaxEnt) [15], Classification and Regression Tree (CART) [16], etc. Moreover, the MaxEnt model is proven to be more accurate compared with other SDMs considering its stable performance on various region levels and sample sizes [17]. Until now, the MaxEnt model has been widely used in the evaluation of endangered plant reserves; used for its potential distribution of invasive plant prediction; and for the impact of climate change on species distribution [18,19,20]. Although the application of SDMs on potential habitats has been reported with single or multiple species, the potential habitat for a whole genus was rarely studied.

Climate is the main factor determining the distribution of plants and the change of plant species distribution is the direct reflection of climate change. According to the Fifth Assessment Report (AR5) of the Intergovernmental Panel on Climate Change (IPCC), the annual average surface temperature of the world will rise by 0.3–4.8 °C by the end of the 21st century [21]. Due to climate change, more than 80% of species might experience changes in relation to diversity, current distribution, and potential habitat [22,23]. Hence, it’s beneficial for plant species conservation to predict the potentially suitable areas under future climate change.

Food security and climate change are major challenges for agriculture in developing countries [24]. Food security and biodiversity conservation are closely related to achieving sustainable food systems on a large scale [25]. Crop yield is an essential factor to evaluate sustainability. The Sustainable Yield Index (SYI) was proposed as an indicator to assess the sustainability of agricultural yield [26], wherein high SYI values and low standard deviation (SD) values indicate higher sustainability [27]. Many studies have shown that the SYI is an important indicator of productivity [28,29].

In the future, it is expected that the demand for food will continue to increase with population growth and declining cultivated land quality. Thus, there is an urgent need to solve the impending trade-off between population, food, and cultivated land. Mitigation of these will depend on the understanding of the potential habitats of buckwheat using predictions in climate change. In this study, buckwheat resources in China were collected and their characteristics were investigated in relation to potential habitats and predicted climate change. Moreover, the SYI values of buckwheat yield in three periods were evaluated and both the development and utilization of buckwheat resources were further discussed.

## 2. Results

### 2.1. Wild Buckwheat Distribution: Current and Future

**Figure 1:**
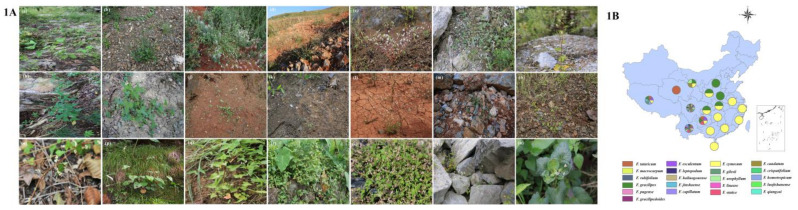
The morphological characteristics and distribution of wild buckwheat species. (**A**) represents the plant morphology and biotope of nineteen wild and two cultivated species: (**a**) *F. tataricum*, (**b**) *F. esculentum*, (**c**) *F. cymosum*, (**d**) *F. statice*, (**e**) *F. jinshaense*, (**f**) *F. qiangcai*, (**g**) *F. pugense*, (**h**) *F. urophyllum*, (**i**) *F. caudatum*, (**j**) *F. gracilipedoides*, (**k**) *F. lineare*, (**l**) *F. leptopodum*, (**m**) *F. gilesii*, (**n**) *F. capillatum*, (**o**) *F. macrocarpum*, (**p**) *F. hailuogouense*, (**q**) *F. gracilipes*, (**r**) *F. luojishanense*, (**s**) *F. rubrifolium*, (**t**) *F. homotropicum*, and (**u**) *F. crispatofolium*. (**B**) represents the distribution of wild buckwheat species in China.

The morphological characteristics of the wild buckwheat species were pretty similar and difficult to distinguish without professional techniques. In addition, most of the wild buckwheat species grew in the steep slope of high mountains, on which the collection was difficult. Practically, multiple wild buckwheat species including *F. callianthum*, *F. gracilipedoides*, *F. lineare*, *F. leptopodum*, *F. gilesii*, and *F. homotropicum* grew in rocky hillside areas. A small part of the wild buckwheat species, including *F. rubrifolium, F. gracilipes*, and *F. luojishanense*, grew close to farmlands. Twenty-one buckwheat species were collected in the field investigations from 2007 to 2017. The pictures of 19 wild buckwheat and two cultivated buckwheat species, as well as their distribution, are illustrated in Figure 1. Except for several regions, the distribution of wild buckwheat was relatively wide in China. At the same time, *F. cymosum*, distributed in 16 regions, was the most widely spread and was followed by *F.gracilipedoides* in 10 regions. In addition, the most abundant occurrence of wild buckwheat was concentrated in southwestern China including Sichuan, Yunnan, and Tibet. Sichuan had the maximum number of wild buckwheat samples with 18 species (Figure 1B). Additionally, their biological and morphological characteristics were evaluated (Table 2 and Table 3).

**Figure 2:**
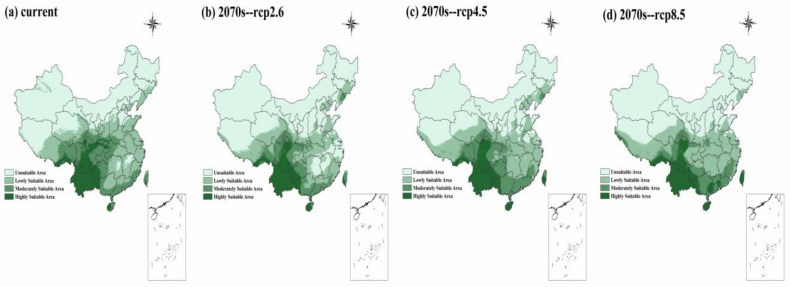
The potentially suitable areas of wild buckwheat species in the current day and in the 2070s.

The AUC value of the training data and test data were 0.934~0.940 and 0.938~0.945 though the MaxEnt model (Supplementary Materials Figure S1), which indicated that the predicted results were credible. In the current climate, the highly suitable area was mainly concentrated in southwest China. The percentage of the three classes of potentially suitable areas (“unsuitable area”, “lowly suitable area”, and “more suitable area”) were 55.44%, 23.86%, and 20.70% of China’s total area. Similar to the current climate, the highly suitable area was concentrated in the southwest of China in three RCPs of the 2070s (Figure 2). The three classes’ potentially suitable area percentages in RCP 2.6 were 63.27%, 20.27%, and 16.46%, and the percentages in RCP 4.5 were 57.73%, 22.95%, and 19.32%, while the percentages in RCP 8.5 were 59.88%, 22.90%, and 17.22% (Supplementary Materials Table S1). Therefore, the unsuitable area was significantly expanded, while the highly suitable area remained the same with a minor change in the future.

### 2.2. Cultivated Buckwheat: History, Development, and Future Distribution

**Figure 3:**
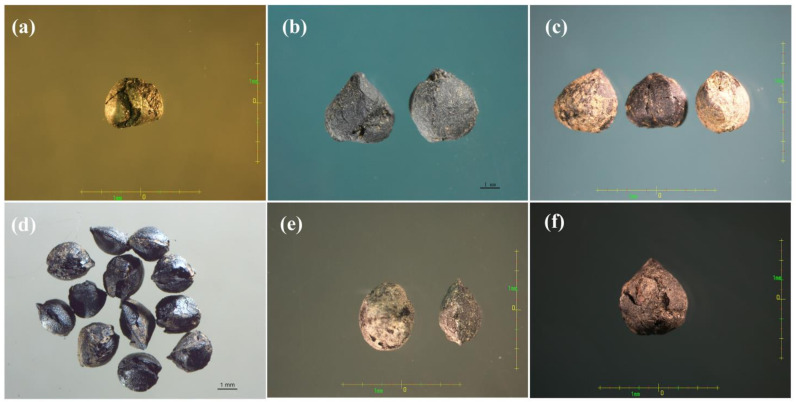
Buckwheat seeds found at different excavation sites from different epochs: (**a**) Chunqiu dynasty, (**b**) Han-Liao-Jin dynasty, (**c**) Han-Liao-Jin dynasty, (**d**) Song dynasty, (**e**) Liao dynasty, and (**f**) Liao-Jin dynasty.

Buckwheat has been cultivated in China for thousands of years. In recent years, massive buckwheat seed samples were unearthed from many ancient excavation sites. So far, the seed fossils were found originating from the Chunqiu dynasty (~2500 years ago), Han-Liao-Jin dynasty (~2000 years ago), Liao-Jin dynasty (~1000 years ago), and Song dynasty (~700 years ago) (Figure 3).

**Figure 4:**
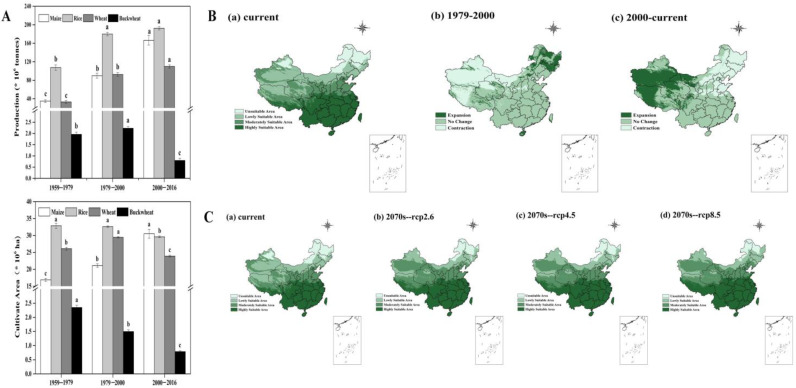
The development of buckwheat cultivation and its potentially suitable areas in the future. (**A**) represents the production and cultivated areas of major crops and buckwheat in 1979–2016; values are means ± SE and different lowercase letters in each crop indicate the significant differences between different patterns (ANOVA followed by the Duncan test, *p* < 0.05). (**B**) represents the changes in buckwheat cultivation and in the potentially suitable areas in 1979–2016. (**C**) represents the potentially suitable areas of buckwheat cultivation in the current day and future.

The production of maize, rice, and wheat was relatively increased, along with time, while buckwheat production increased by 13.89% in 1979–2000 and decreased by 64.33% in 2000–2016, compared with the previous period. The cultivated areas of maize was significantly increased by 80.89% from 1959 to 2016. Meanwhile, the buckwheat cultivated areas were decreased by 66.37% compared with the previous period (Figure 4A). In the three periods, compared with other countries or regions, the buckwheat SYI value in China was the lowest, while the CV index was the highest (Table 4). In addition, the buckwheat SYI value in Europe and South Korea increased by 29.41% and 61.36%, respectively. However, the buckwheat SYI value in Japan decreased by 18.18% from 1959 to 2016. Among the four countries or regions, the buckwheat SYI value in Japan was the highest (0.77) from 1959 to 1979, while the buckwheat SYI value in South Korea was the highest (0.71) from 2000 to 2016.

The expansion of potentially suitable areas occurred in the northeast of China and the contraction occurred in the northwest of China during 1979–2000. In contrast, the expansion occurred in the northwest while the contraction occurred in the northeast from 2000 to the present day (Figure 4B).

Most areas of China were suitable for buckwheat growth, except part of the northeast, in the current day and future (Figure 4C). Currently, the percentage of the three classes as potentially suitable areas for cultivating buckwheat (“unsuitable area”, “lowly suitable area”, and “more suitable area”) were 13.98%, 32.10%, and 53.91% of China’s total area. In the future, the three classes potentially suitable for cultivation in RCP 2.6 were 9.30%, 21.67%, and 69.03%, and in RCP 4.5 were 9.58%, 23.22%, and 67.20%, while in RCP 8.5 they were 7.34%, 21.38%, and 71.28% (Supplementary Materials Table S2). Thus, the unsuitable area was decreased tremendously and the highly suitable area was increased.

## 3. Discussion

### 3.1. Buckwheat Distribution and Place of Origin

Buckwheat belongs to the *Polygonaceae* family, which includes nineteen wild species and two cultivated species. Initially, the Swiss plant taxonomist De Candolle [30] proposed that buckwheat originated in Siberia and northern China. However, Nakao [31] suggested that southern China might be the origin of buckwheat rather than Siberia or northern China considering large amounts of wild buckwheat relatives were distributed in southern China. Then, since the 1980s, many Chinese buckwheat researchers considered southwestern China as the original center based on historiography and the abundance of wild relatives [32,33,34]. Later, Tsuji and Ohnishi suggested that the eastern part of Tibet and the joint area of Yunnan and Sichuan were the places of origin for cultivated Tartary buckwheat using different types of analysis, such as morphology, reproductive biology, isoenzyme analysis, RAPD, and AFLP [2,3,4]. Buckwheat scientists around the world widely accepted that the southwest of China was not only the center of diversity of buckwheat but also the origin of cultivated buckwheat. In this research, similar results were obtained concerning that the southwest of China represented the diversity center of buckwheat due to the distribution of wild buckwheat species (Figure 1B). Additionally, there were more unearthed buckwheat seeds from the ancient excavation sites as evidence to prove the origin of buckwheat. This showed that buckwheat cultivation might have been practiced nearly 2500 years ago (Figure 3).

### 3.2. Development of Cultivated Buckwheat

Buckwheat production decreased in 2000–2016, perhaps due to the reduction in cultivated areas within the same three periods (Figure 4A). It was found that the yield of buckwheat was decreased in 2000–2016, which might largely depend on a nutrient deficiency of buckwheat cultivated in barren soil caused by the encroachment of major crop-cultivation areas. However, the production and cultivated areas of major crops (maize, wheat, etc.) were increased during the three periods. Hence, it’s considered that the decrease in the production of buckwheat was due to the decrease of buckwheat cultivated areas, which were affected by the increase of major crop-cultivated areas. In contrast, there were other changes in the development process of buckwheat cultivation in Europe, Japan, and South Korea (Supplementary Materials Figure S2). In Europe, the production of buckwheat and major crops were all increased. The production and cultivation areas of buckwheat were increased, while the major crops were decreased in Japan. Nevertheless, both the production and cultivation areas of buckwheat were decreased in South Korea. The potentially suitable areas of cultivated buckwheat in China during 1959–1979 were higher than in the other two periods, which might have been caused by its tolerance to barren conditions, therefore it received more attention in a period of low productivity. In addition, the potentially suitable areas of cultivated buckwheat during 2000–2016 were more improved than during 1979–2000, which might have been the result of the green revolution in China.

From 1959 to 2016, the buckwheat SYI value in China was the lowest over the three periods in comparison to other countries and regions, illustrating that the sustainability of the cultivated buckwheat yield in China was low (Table 4). In addition, the CV index of buckwheat yield in China was the highest over the three periods, indicating unstable buckwheat yield in China. This might be closely related to the barren growth conditions of buckwheat, which made it difficult to maintain a sustained high yield. This proved that there was a significant correlation between crops’ SYI and soil active organic matter [29,35]. More evidence showed that nutrient availably could improve buckwheat yield [36,37]. Therefore, improving soil fertility and strengthening cultivation measures were important to effectively improve buckwheat yield and its sustainability.

Despite the lower sustainability of buckwheat yield in China, the potentially suitable areas of cultivated buckwheat showed an obvious expansion (Figure 4C) and the result was consistent with others [20,38]. With the expansion of the suitable areas, buckwheat production would be increased on the basis of ensuring soil fertility, which could enhance the sustainability of buckwheat production under climate change. Thus, more intense promotion of buckwheat cultivation could fill the food gap caused by crops that would be unsuitable for growth due to climate changes in the future.

### 3.3. Wild Resources Protection under Climate Change

With global warming, the species’ potentially suitable areas would vary in change, such as by expanding, contracting, or not experiencing much change. Yi et al. [20] suggested that the habitat of *Homonoia riparia* Lour in Yunnan Province would expand in the future. Under the future climate change scenario, *Tylophora hirsuta* was predicted to lose most of its habitats in northern Punjab and in parches from lower peaks of Galliat, Zhob, Qalat, etc., and the MaxEnt model might be applied to other rare Asclepiad species, especially those under constant extinction threat [39]. However, the suitable habitat areas of *Stipa purpurea* tended to increase from the 1990s to 2050s, while a decline from the 2050s to 2070s is expected [18]. According to the results, in the 2070s, the unsuitable areas for wild buckwheat will increase, while the suitable areas will decrease by 6.7% and 16.8% under RCP 4.5 and RCP 8.5, respectively (Figure 2). Similar to this result, Luitel et al. [40] reported that suitable habitats of buckwheat will decrease by 8.2% and 8.3% under RCP 4.5 and RCP 8.5 in 2070, respectively. They were concerned that the diversity of wild buckwheat species would decrease in the future. What should be noted is that some narrowly distributed species might be endangered by extinction, such as *F. hailuogouense*, *F. rubifolium*, *F. lineare*, and *F. crispatofolium*. In particular, *F. hailuogouense* was distributed only in the snow line on Minya Konka, which means it would disappear once the snow melts. Not only due to climate change, but the lack of awareness of buckwheat and excessive human activity might also reduce the diversity of the wild buckwheat species. Accordingly, more effective conservation measures should be implemented to protect wild buckwheat species. Although *F. cymosum*, as a second-class national protected plant, has been conserved to a certain extent in China, the protection of other wild buckwheat species is still very limited. Therefore, both on-site (in situ) conservation and off-site (ex situ) preservation should be performed simultaneously, including making protective policies, establishing nature reserves, etc. Then, the introduction and domestication of wild buckwheat species would strengthen. Meanwhile, the gene bank should be established to conserve wild buckwheat genetic resources.

### 3.4. Wild Buckwheat Utilization

**Figure 5:**
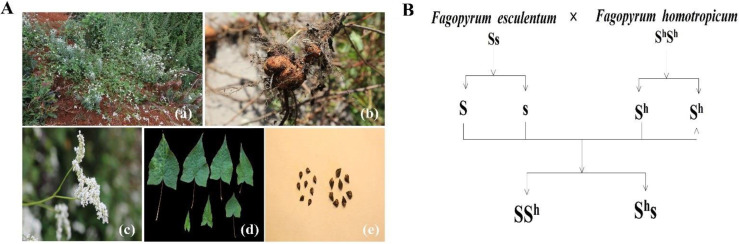
The utilization of *F. cymosum* and *F. homotropicum*. (**A**) represents the morphological characteristics of *F. cymosum*. (**B**) represents a flowchart of the wide hybridization between *F. homotropicum* and *F. esculentum*.

Wild buckwheat species are rich in protein and have both essential amino acids for human beings (such as threonine, methionine, valine, leucine, phenylalanine, isoleucine, lysine, and tryptophan) and therapeutic compounds [5]. Thus, wild buckwheat should be reasonably developed and utilized on the basis of adequate protection. Initially, buckwheat was utilized in products such as tea, sprouts, cakes, bread, biscuits, vermicelli, and flour [5]. Although wild buckwheat species remain underutilized, the products made from cultivated buckwheat species are relatively abundant. A variety of buckwheat products around the world are summarized in Table 5, in which food categories with the highest share of the market within buckwheat products include unprocessed cereals (33.8%), biscuits (18.1%), pasta (10.6%), and breakfast cereals (9.4%), based on a survey of 160 buckwheat-containing products in the Slovenian market [41]. However, more buckwheat products with higher added values and better medicinal benefits have been developed with an in-depth understanding of the nutritional value and medicinal value of buckwheat. In this context, *F. cymosum* played an important role in buckwheat utilization due to its excellent medicinal properties; the root, leaf, stem, flower, and grain of *F. cymosum* (Figure 5A) are all rich in bioflavonoids including rutin and quercetin [5]. It was reported that the extracts of *F. cymosum* possessed anti-tumor, anti-oxidation, anti-inflammatory, anti-aging, hypoglycemic, anti-allergic, and anti-fatigue effects, among others [42,43,44]. Currently, the root, stem, leaves, and flower of *F. cymosum* are used for the production of tablets for a mixture for clinical treatment, such as in the Weimaining capsule. The ethanol extract of *F. cymosum* roots had an effect on the treatment of 71% of lung cancer patients [45,46]. Meanwhile, *F. cymosum* and *F. homotropicum* are used in cross-breeding due to their pharmaceutical applications. The wild buckwheat *F. homotropicum*, which is similar to *F. esculentum* in morphology, could be used for hybridization because of its homostyly and self-fertility features (Figure 5B).

Due to its high environmental adaptability, wild buckwheat has a high potential to expand utilization compared with other crops. It was showed that buckwheat might be a high UV-B tolerance crop considering its high-altitude habitat [45]. Meanwhile, buckwheat is relatively tolerant to high temperatures [46]. In addition, buckwheat was adapted to barren soil compared with other cereals [45,47]. Currently, most studies concentrated on the effect of abiotic stress on cultivated buckwheat (*F. esculentum* and *F. tataricum*). However, it should be noted that wild buckwheat species might have a higher environmental adaptability considering their harsher habitats. Therefore, the research on the response of wild buckwheat to abiotic stress needs to be enhanced.

In addition, there is another aspect that enhances the utilization of wild buckwheat, namely breeding. Compared with other wild species, *F. homotropicum* is superior in terms of wide hybridization due to its self-fertility. Since 1995, it has been reported that successful inter-specific hybridization between diploid *F. esculentum* (2*n* = 16) and diploid *F. homotropicum* (2*n* = 16) has occurred [47,48,49]. Interspecific crosses could be used for the improvement of the agricultural traits of cultivated buckwheat.

## 4. Materials and Methods

### 4.1. Buckwheat Resources by Field Investigation

The field investigation of the buckwheat species was carried out from 2007 to 2017 in China. The study was carried out on nineteen wild species including *F. cymosum*, *F. hailuogouense*, *F. qiangcai*, *F. gracilipes*, *F. caudatum*, *F. pugense*, *F. leptopodum*, *F. luojishanense*, *F. rubifolium*, *F. jinshaense*, *F. gilesii*, *F. urophyllum*, *F. lineare*, *F. gracilipedoides*, *F. statice*, *F. crispatifolium*, *F. macrocarpum*, *F. capillatum*, and *F. homotropicum*, and two cultivated species, *F. esculentum* and *F. tataricum*, were also included.

The plant characteristics included karyotype, growth period, life cycle, plant height, plant shape, flower color, inflorescence shape, style type, pollination, perianth shape, perianth length, leaf shape, leaf width, leaf length, leaf hair, stem hair, seed shape, and seed size. Chromosomes’ ploidy was measured by karyotype analysis (OLYMPUS AX80, Olympus Corporation Ltd., Tokyo, Japan), while plant height, leaf width, and leaf length were measured as an average of ten random buckwheat plants of each entry with three repetitions. The perianth length and seed size were measured with three repetitions by a microscope (Leica M165C, Leica Microsystems Ltd., Wetlzar, Germany). The growth period, life cycle, and pollination were scored on potted planting. The plant shape, flower color, inflorescence shape, style type, perianth shape, leaf shape, leaf hair, stem hair, and seed shape were defined by classical taxonomy. The location of 19 wild buckwheat species were collected (Supplementary Materials Table S3). The morphological photos of wild resources were taken by Canon EOS 6D and modified by PhotoShop CS5. The date of ancient buckwheat seeds was identified by radiocarbon ^14^C dating [50].

### 4.2. Data Collection

The data of the buckwheat yield, production, and cultivated areas were collected from the Food and Agriculture Organization (FAO) database (http://www.fao.org/faostat/zh/#data/QC (accessed on 17 October 2018)). The occurrence records of wild buckwheat were collected from the field investigation from 2007 to 2017 and from the Chinese Virtual Herbarium databases (CVH, http://www.cvh.ac.cn (accessed on 17 October 2018)). Where the geographic coordinates were lacking in the occurrence records, the Google Earth map (http://www.google.cn/maps (accessed on 17 October 2018)) was used for locating geographic coordinates. In total, 1,237 occurrence records were obtained after the deletion of duplicates. The occurrence records of cultivated buckwheat in the different periods were obtained from the first national buckwheat resources survey (1959–1979), the second national buckwheat resources survey (1979–2000), and the database of the Chinese Academy of Agricultural Sciences (2000–2016). The occurrence records in different periods totaled to 396, 295, and 139 records, respectively; after the deletion of duplicates.

The current climate conditions of 19 bioclimatic variables (Supplementary Materials Table S4) at 2.5 arc-minute spatial resolution were downloaded from WorldClim version 2.0 (http://worldclim.org (accessed on 25 September 2018)). The average data were included for the years 1970–2000 [51]. The future climate conditions of 19 bioclimatic variables in the 2070s at 2.5 arc-minute spatial resolution were downloaded from the Agriculture and Food Security database (CCAFS, http://www.ccafs-climate.org (accessed on 25 September 2018)). Three Representative Concentration Pathways (RCP 2.6, RCP 4.5, and RCP 8.5) were chosen under the General Circulation Model (GCM) of the Beijing Climate Center Climate System Model version 1.1 (BCC-CSM1.1 model). The three RCPs represented different values of the radiative forcing level between the preindustrial era and the year 2100 [52,53]. RCP 2.6 represented the low scenario with a peak in radiative forcing at 3 W/m^2^ before 2100 and then declined [54]. RCP 4.5 represented the medium scenario without an overshoot pathway to 4.5 W/m^2^ at stabilization after 2100 [55]. RCP 8.5 represented the high scenario in which rising radiative forcing pathway led to 8.5 W/m^2^ in 2100 [56]. Due to different species of buckwheat being influenced by different variables, the bioclimatic variables were not selected to avoid missing important variables.

### 4.3. The Sustainability of the Buckwheat Yield

The sustainability of the cultivated buckwheat yield over the three periods was analyzed by SYI. The stability of the cultivated buckwheat yield over the three periods was also analyzed by the coefficient variation (CV) and the calculation formula is as follows:

(1) $$\mathrm{SYI}=\left(Y-\sigma_{n-1}\right)/Y_{max}$$

(2) $$\mathrm{CV}=\sigma_{n-1}/Y$$

where Y (kg/hm^2^) is the mean yield per period, $\sigma_{n-1}$ is the standard deviation, and $Y_{max}$ (kg/hm^2^) is the maximum yield per period.

### 4.4. MaxEnt Model

The MaxEnt model was a density estimation and species distribution prediction model based on the maximum entropy theory [57]. The principle of this model was to admit the known factors without any hypotheses regarding the unknown factors and select the distribution with the maximum entropy as the optimal distribution; thus, the credible prediction was obtained [15,58].

The occurrence records of buckwheat and bioclimatic variables were loaded into the MaxEnt model 3.3.3 (http://www.cs.princeton.edu (accessed on 17 October 2018)) [15], in which 75% of the sample data was selected as training data randomly and the other 25% of the sample data was used as verification data. The significance of environmental variables was obtained by the jackknife method and the remaining parameters were the default settings of the program. The receiver operating characteristic curve (ROC) was used to check the accuracy of the MaxEnt model. The ROC curve is based on the accuracy of the threshold-independent evaluation with the false positive rate (specificity) as the abscissa and the true positive rate (sensitivity) as the ordinate (Webb and Kai, 2005). The AUC (area under ROC) indicated the performance of the model prediction: AUC > 0.9 for “excellent”, 0.8 < AUC < 0.9 for “good”, 0.7 < AUC < 0.8 for “fair”, 0.6 < AUC < 0.7 for “poor”, and AUC < 0.5 for “fail” [59,60]. The result of the model operation was an ASC file. We converted the ASC file to a raster file and extracted it by the China map (1:4,000,000). Then, the extracted results were dealt with “Natural Breaks” and reclassified to four classes including “unsuitable area”, “lowly suitable area”, “moderately suitable area”, and “highly suitable area”. The “more suitable area” was defined as the sum of the “moderately suitable area” and “highly suitable area”. The above processes were carried out using the Arctoolbox of ArcGIS 10.0 (Esri, CA, USA).

### 4.5. Statistical Analysis

Data were analyzed by one-way ANOVA with the Duncan test in SPSS 19.0, mean values were compared by the least significant difference, and *p* < 0.05 was considered significant. The pictures were processed by the software Origin 9.0.

## 5. Conclusions

The morphological characteristics and geographical distribution of 21 buckwheat species in China were analyzed through field investigation. It was found that the potentially suitable areas of wild buckwheat will decrease in the future by using the MaxEnt model, which might lead to a decrease of buckwheat diversity. In particular, narrowly distributed wild buckwheat species including *F. hailuogouense*, *F. rubifolium*, *F. lineare*, and *F. crispatofolium* will experience high risks of extinction as their habitats will be threatened by climate change. However, the potentially suitable areas of cultivated buckwheat was expected to expand, which indicated that cultivated buckwheat had a stronger adaptability to climate change. Meanwhile, the sustainability of the cultivated buckwheat yield was low according to the SYI value over three periods. In view of this, we believe that buckwheat resources should be protected reasonably. More measures, such as making protective policies and establishing both nature reserves and gene banks for buckwheat resources, would be promising ways to achieve its protection. Then, further development and utilization of buckwheat resources should be carried out.

## Figures and Tables

**Table 1:** The 21 species of genus Fagopyrum.

Number	Scientific Name	Number	Scientific Name
**Wild species**		**Wild species**	
1	*Fagopyrum cymosum* (Trevir.) Meisn	12	*Fagopyrum urophyllum* (Bureau & Franch.) H. Gross
2	*Fagopyrum hailuogouense* J.R. Shao, M.L. Zhou & Qian Zhang	13	*Fagopyrum lineare* (Sam.) Haraldson
3	*Fagopyrum macrocarpum* Ohsako & Ohnishi	14	*Fagopyrum gracilipedoides* Ohsako & Ohnishi
4	*Fagopyrum qiangcai* D.Q. Bai	15	*Fagopyrum statice* Gross
5	*Fagopyrum capillatum* Ohnishi	16	*Fagopyrum crispatifolium* J.L. Liu
6	*Fagopyrum gracilipes* (Hemsl.) Dammer	17	*Fagopyrum caudatum* (Sam.) A.J.Li
7	*Fagopyrum pugense* T. Yu	18	*Fagopyrum gilesii* (Hemsl.) Hedberg
8	*Fagopyrum homotropicum* Ohnishi	19	*Fagopyrum jinshaense* Ohsako & Ohnishi
9	*Fagopyrum leptopodum* (Diels) Hedberg	**Cultivated species**	
10	*Fagopyrum luojishanense* J.R. Shao	20	*Fagopyrum esculentum* Moench
11	*Fagopyrum rubifolium* Ohsako & Ohnishi	21	*Fagopyrum tataricum* (L.) Gaertn

**Table 2:** The biological characteristics of wild buckwheat.

Scientific Name	Ploidy	Growth Period	Life Cycle	Style Type	Pollination
*F. esculentum*	D	May to August	A	HE	CP
*F. tataricum*	D	May to September	A	HO	SP
*F. qiangcai*	D	July to November	A	HE	CP/SP
*F. macrocarpum*	--	July to November	A	HE	CP/SP
*F. rubifolium*	D/T	July to November	A	HO	SP
*F. gracilipes*	T	June to November	A	HO	SP
*F. pugense*	D	July to November	A	HO	CP/SP
*F. luojishanense*	D	June to November	A	HO	SP
*F. jinshaense*	D	July to November	A	HE	CP
*F. capillatum*	D	July to November	A	HE	CP
*F. gracilipedoides*	D	July to November	A	HE	CP
*F. gilesii*	D	July to November	A	HE	CP
*F. urophyllum*	D	April to November	P	HE	CP
*F. lineare*	D	August to November	A	HE	CP
*F. statice*	D	July to November	P	HE	CP
*F. caudatum*	D	June to November	A	HE	CP
*F. crispatofolium*	T	July to November	A	HO	SP
*F. homotropicum*	D/T	July to October	A	HO	SP
*F. cymosum*	D/T	May to December	P	HE	CP
*F. leptopodum*	D	June to November	A	HE	CP
*F. hailuogouense*	D	May to August	P	HO	SP

Note: D = diploid and T = tetraploid in Ploidy column; A = annual and P = perennial in Life Cycle column; HE = heterostyly and HO = homostyly in Style Type column; and CP = cross-pollination and SP = self-pollination in Pollination column.

**Table 3:** The morphological characteristics of wild buckwheat.

Scientific Name	Plant Height (cm)	Plant Shape	Flower Color	Inflorescence Shape	Perianth Shape	Perianth Length (mm)	Leaf Shape	Leaf Width (cm)	Leaf Length (cm)	Leaf Hair	Stem Hair	Seed Shape	Seed Size (mm)
*F. esculentum*	60.0–150.0	P/SE	P/R	CR	E	3.0–4.0	HA, HS, OVT	2.0–5.0	2.5–7.0	G	G	T	4.0–6.0
*F. tataricum*	80.0–160.0	E	LG	CO	OV	2	HA, HS, T	2.8	2.7	G	G	OT	1.5–2.5
*F. qiangcai*	15.0–70.0	C/O	W/P	R/CO	E/OV	3.5–4.0	HS, OV, T	1.2–4.0	1.2–4.5	G	G	T	2.0–5.0
*F. macrocarpum*	5.0–75.0	C	W/P	CO	E	--	HA, HS	1.2–3.0	2.0–3.5	LH	G	--	--
*F. rubifolium*	5.0–115.0	C/O	P	R	O	--	HA, HS	1.0–3.6	2.5–4.2	H	H	T	1.5–2.5
*F. gracilipes*	20.0–70.0	E	W/P	R	E	2.0–2.5	OVT	1.5–3.0	2.0–4.0	LH	G	OT	3
*F. pugense*	17.0–70.0	E/O/P	W/P	R	E/OV	1.3–2.0	HS, OV, OVT	1.2–4.6	1.7–5.5	H	H	OT	1.8–2.5
*F. luojishanense*	40.0–70.0	C/SE	W/P	R	E	1.3–2.0	OV, T	1.2–5.1	1.7–6.0	LH	LH	OT	1.8–2.5
*F. jinshaense*	14.2-31.8	E	W/P	SR	E	--	HA, HS, T, S	0.2–1.4	0.5–1.4	G	G	OT	<1.5
*F. capillatum*	60.0–150.0	E	W/P	R	OV	--	HA, HS, OV	0.4–2.0	0.4–3.2	H	H	OT	1.5–2.0
*F. gracilipedoides*	20.0–50.0	SE	W/P	R	E	--	HA, HS, OV	0.4–2.0	0.4–3.2	LH	LH	OT	2.0–3.0
*F. gilesii*	16.0–44.0	SE	W/P	CA	E	2.0–2.5	HS	0.6–2.5	0.9–3.5	H	G	OT	3.0–4.0
*F. urophyllum*	180.0–225.0	E	W/P	CC	E	2.0–3.0	HS, AS	0.9–7.2	1.3–9.8	H	G/LH	OT	3.0–4.0
*F. lineare*	22.0–40.0	E	W	R	E	1.5	L, HA	0.1–1.5	0.5–2.2	G	G	T/ET	2
*F. statice*	40.0–50.0	E	W/P	CO	E	1.0–1.5	HA, L, T, OV	1.5–3.0	2.0–3.0	G	G	T/OT	2.0–2.5
*F. caudatum*	27.0–50.0	O/P	W	R	E/OB	2.0 -2.5	S	4.0–10.0	1.0-3.0	G	G	ET	3
*F. crispatofolium*	44.0–88.0	E/O/P	W	R	E/OB	1.8-2.0	HS, OV	1.5–6.8	2.0–7.7	H	LH	T	4.0–5.0
*F. homotropicum*	60.0–130.0	E/O	P	R	E	2.0–2.5	HA, HS, OVT	1.5–4.5	1.5–6.0	G	G	OT	2.5–3.0
*F. cymosum*	50.0–300.0	C/E	W	CO	E/OV	3.5–4.0	HA, OVT	6.1–9.4	5.2–8.3	G/H/LH	G/H/LH	CT/ET	6.0–8.0
*F. leptopodum*	6.0–60.0	C/E	W/P	R	E	1.5–2.5	OV, T	1.0–1.5	1.5–2.5	G	G	OT	2
*F. hailuogouense*	30.0–70.0	E/O	W/P	CO	OV	1.8–2.0	HS, OV	1.8–5.3	2.2–5.8	G	G	OT	2.5–3

Note: C = creeping, E = erect, O = oblique, P = prostrate, and SE = semierect in Plant Shape column; P = pink, R = red, W = white, and LG = light green in Flower Color column; CA = capitulum, CO = corymb, CC = conical corymb, R = raceme, CR = conical raceme, and SR = spicate raceme in Inflorescence Shape column; E = elliptical, OB = obovoid, and OV = oval in Tepal Shape column; L = linear, HA = hastate, HS = heart-shaped, OV = oval, OVT = oval triangle, T = triangular, S = sagittate, and AS = auricular sagittate in Leaf Shape column; G = glabrous, H = hairy, and LH = less hair in Leaf Hair and Stem Hair column; and T = three pyramid, CT = conical three prism, HT = hastate three pyramid, OT = oval three pyramid, and ET = elliptic three prism in Seed Shape column.

**Table 4:** The sustainability of the cultivated buckwheat yield from 1959 to 2016.

	China		Europe		Japan		South Korea	
	SYI	CV	SYI	CV	SYI	CV	SYI	CV
1959–1979	0.37	0.35	0.51	0.23	0.77	0.11	0.44	0.31
1979–2000	0.61	0.18	0.64	0.17	0.72	0.14	0.72	0.09
2000–2016	0.40	0.31	0.66	0.16	0.63	0.18	0.71	0.10

**Table 5:** International buckwheat products.

China	Europe	Japan	South Korea
Buckwheat tea	Unprocessed cereals	Buckwheat noodle	Buckwheat noodle
Buckwheat pillow	Breakfast cereals	Buckwheat honey	Buckwheat pancake
Buckwheat noodle	Pasta	Buckwheat tea	Buckwheat pillow
Buckwheat flour	Biscuit	Buckwheat pillow	Buckwheat bouquet
Buckwheat honey	Bread	Buckwheat rice	Buckwheat tea
Buckwheat cake	Soup	Buckwheat seasoning	Buckwheat flour
Buckwheat liquor	Cereal bars	Buckwheat flour	Buckwheat cake
	Baby food		
	Cakes, muffins, and pastry		
	Meat alternatives		
	Coffee and tea		
	Honey and syrups		
	Chocolate and sweets		
	Canned fish and seafood		
	Yoghurt products		
	Processed meat and derivatives		

Note: the data was collected from the website of the Ministry of Commerce of People’s Republic of China and through personal communication.

## Data Availability

Not applicable.

## Supplementary Materials

The following are available online at https://www.mdpi.com/article/10.3390/plants10102081/s1, Table S1: The percentages of wild buckwheat species’ potentially suitable areas in China; Table S2: The percentages of potentially suitable areas for cultivated buckwheat in China; Table S3: The collection locations of 19 wild buckwheat species; Table S4: The meaning of 19 bioclimatic variables; Figure S1: The results of the AUC of wild buckwheat in the MaxEnt model; and Figure S2: The development progress of buckwheat cultivation in Europe, Japan, and South Korea.
