# Supplementary material for: The Distribution and Sustainable Utilization of Buckwheat Resources under Climate Change in China
Source: Plants (Basel). 2021 Sep 30;10(10):2081. doi: 10.3390/plants10102081 (PMC8538749; doi:10.3390/plants10102081)
Supplement: Supplementary file 1 [file plants-10-02081-s001.zip › plants-1353770-supplementary.pdf]

Table S1. The percentage of wild buckwheat species potentially suitable area in China

Potentially suitable area	Current	2070s		
		RCP 2.6	RCP 4.5	RCP 8.5
Unsuitable area	55.44%	63.27%	57.73%	59.88%
Lowly suitable area	23.86%	20.27%	22.95%	22.90%
Moderately suitable area	12.63%	8.96%	11.25%	8.76%
Highly suitable area	8.07%	7.50%	8.08%	8.47%
More suitable area	20.70%	16.46%	19.32%	17.22%

Note: the percentage = the potential area in four classes / the total area of China

Table S2. The percentage of cultivated buckwheat potentially suitable area in China

Potentially suitable area	Current	2070s		
		RCP 2.6	RCP 4.5	RCP 8.5
Unsuitable area	13.98%	9.30%	9.58%	7.34%
Lowly suitable area	32.10%	21.67%	23.22%	21.38%
Moderately suitable area	24.57%	35.21%	33.03%	33.78%
Highly suitable area	29.35%	33.83%	34.16%	37.50%
More suitable area	53.91%	69.03%	67.20%	71.28%

Note: the percentage = the potential area in four classes / the total area of China

Table S3. The collection locations of 19 wild buckwheat species

Scientific Name	Location
F. qiangcai	Tibetan Qiang Autonomous Prefecture of Ngawa, Sichuan province
F. macrocarpum	Tibetan Qiang Autonomous Prefecture of Ngawa, Sichuan province
F. rubifolium	Tibetan Qiang Autonomous Prefecture of Ngawa, Sichuan province
F. gracilipes	Liangshan Yi Autonomous Prefecture, Sichuan province
F. pugense	Liangshan Yi Autonomous Prefecture, Sichuan province
F. luojishanense	Liangshan Yi Autonomous Prefecture, Sichuan province
F. jinshaense	Lijiang city, Yunnan province
F. capillatum	Lijiang city, Yunnan province
F. gracilipedoides	Lijiang city, Yunnan province
F. gilesii	Diqing Tibetan Autonomous Prefecture, Yunnan province
F. urophyllum	Lijiang city, Yunnan province
F. lineare	Dali city, Yunnan province
F. statice	Honghe Hani and Yi Autonomous Prefecture, Yunnan province
F. caudatum	Lijiang city, Yunnan province
F. crispatofolium	Liangshan Yi Autonomous Prefecture, Sichuan province
F. homotropicum	Lijiang city, Yunnan province
F. cymosum	Liangshan Yi Autonomous Prefecture, Sichuan province
F. leptopodum	Ya'an city, Sichuan province
F. hailuogouense	Ganzi Tibetan Autonomous Prefecture, Sichuan province

Table S4. The meaning of 19 bioclimatic variables

Bioclimatic variables	Meaning	Bioclimatic variables	Meaning
Bio1	Annual Mean Temperature [°C]	Bio11	Mean Temperature of Coldest Quarter [°C]
Bio2	Mean Diurnal Range (Mean of monthly (max temp - min temp)) [°C]	Bio12	Annual Precipitation [mm]
Bio3	Isothermality (Bio2/Bio7) (* 100) [°C]	Bio13	Precipitation of Wettest Month [mm]
Bio4	Temperature Seasonality (standard deviation *100) [°C]	Bio14	Precipitation of Driest Month [mm]
Bio5	Max Temperature of Warmest Month [°C]	Bio15	Precipitation Seasonality (Coefficient of Variation) [mm]
Bio6	Min Temperature of Coldest Month [°C]	Bio16	Precipitation of Wettest Quarter [mm]
Bio7	Temperature Annual Range (Bio5-Bio6) [°C]	Bio17	Precipitation of Driest Quarter [mm]
Bio8	Mean Temperature of Wettest Quarter [°C]	Bio18	Precipitation of Warmest Quarter [mm]
Bio9	Mean Temperature of Driest Quarter [°C]	Bio19	Precipitation of Coldest Quarter [mm]
Bio10	Mean Temperature of Warmest Quarter [°C]		

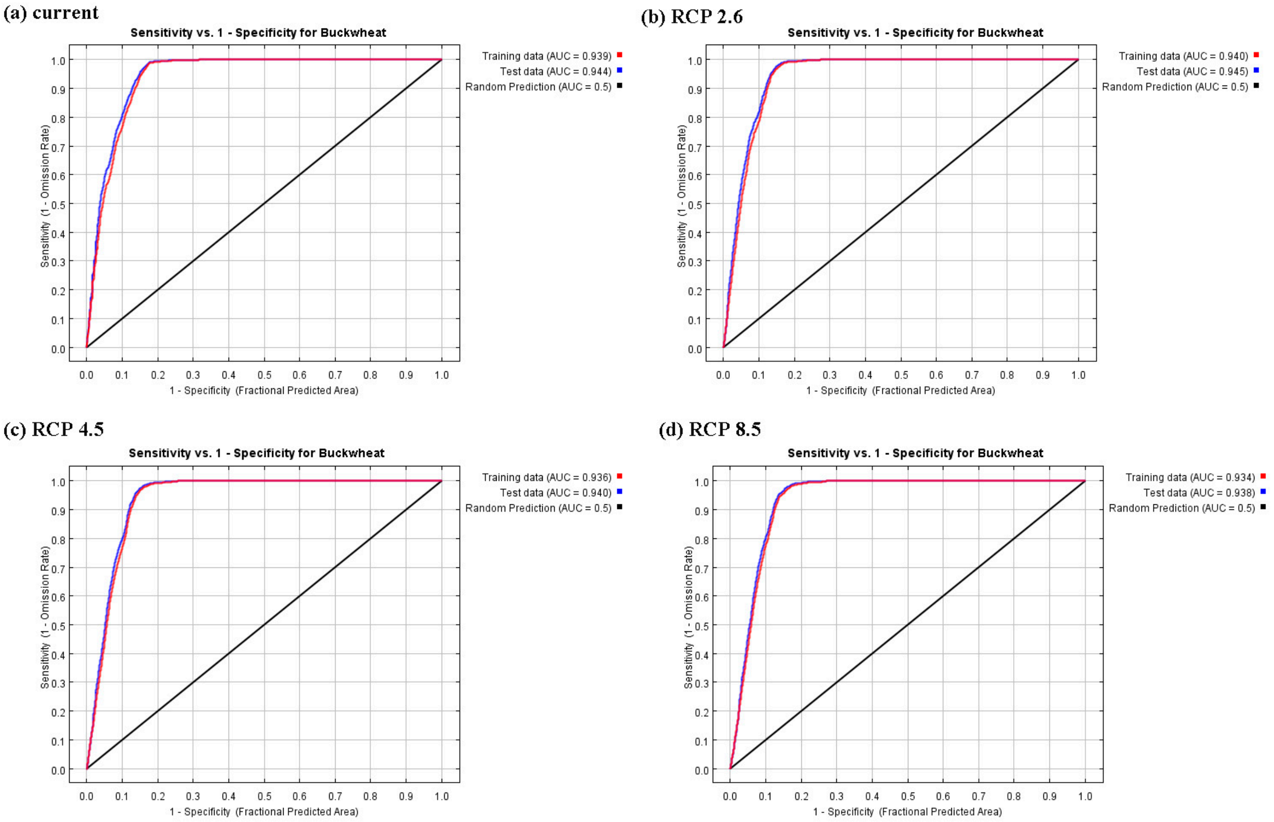

Figure S1. The results of AUC of wild buckwheat in MaxEnt model

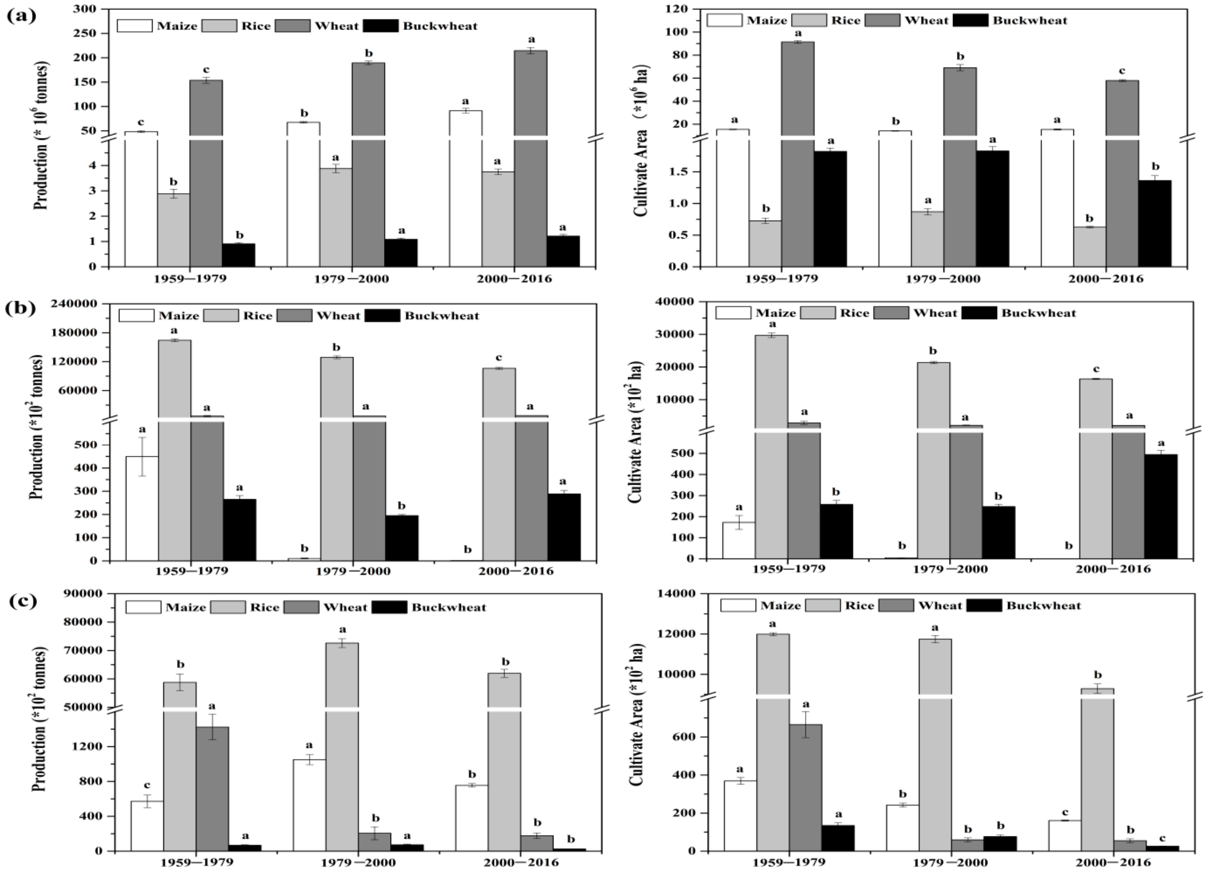

Figure S2. The development progress of buckwheat cultivation in Europe, Japan and South Korea. (a) Europe, (b) Japan, (c) South Korea. Values are means ± SE, different lowercase letters in each crop indicate the significant differences between different patterns (ANOVA followed by Duncan test, $p < 0.05$).
